# Inflammatory Myofibroblastic Tumor of the Bladder Following Intravesical Bacillus Calmette-Guérin Therapy: A Case Report

**DOI:** 10.7759/cureus.117116

**Published:** 2026-09-28

**Authors:** Mucahit Alan, Selçuk Kaba, Esin Yiğitbaşı Kılıç, Serkan Akan

**Affiliations:** 1 Department of Urology, Fatih Sultan Mehmet Eğitim Ve Araştırma Hastanesi, İstanbul, TUR; 2 Department of Pathology, Fatih Sultan Mehmet Eğitim Ve Araştırma Hastanesi, İstanbul, TUR

**Keywords:** bacillus calmette-guérin (bcg) immunotherapy, bladder ca, inflammatory myofibroblastic tumor (imt), non-muscle-invasive bladder cancer(nmibc), postoperative spindle cell nodule

## Abstract

Inflammatory myofibroblastic tumor (IMT) is a rare mesenchymal neoplasm of the bladder and frequently mimics bladder cancer recurrence. We present a rare case of bladder IMT developing following intravesical Bacillus Calmette-Guérin (BCG) therapy.

A 74-year-old male patient was treated with transurethral resection (TURBT) for low-grade (LG) Ta non-muscle-invasive bladder cancer (NMIBC). Subsequently, no recurrence was detected during the follow-up cystoscopies carried out at three and 12 months. During an 18-month follow-up cystoscopy, multiple 4-5 mm papillary lesions of LG NMIBC recurrence were detected, leading to TURBT followed by intravesical BCG therapy. Three weeks after the end of induction therapy, the patient presented with painless macroscopic hematuria. Cystoscopy revealed a solid lesion measuring approximately 120 × 90 mm, filling most of the bladder lumen and differing morphologically from the previous papillary tumors; apart from a single 10-mm stalk, it had no connection to the bladder mucosa. The lesion was resected. Histopathological examination was consistent with IMT.

IMT should be considered in the differential diagnosis of atypical bladder lesions showing spindle-cell morphology in patients who have received intravesical BCG therapy. Although a definitive causal relationship between BCG and IMT has not been established, the short interval between the completion of BCG induction therapy and the diagnosis of IMT in this case suggests a possible association. Further research is warranted to clarify this potential relationship.

## Introduction

Inflammatory myofibroblastic tumor (IMT) is a rare mesenchymal neoplasm of the bladder, in contrast to the predominantly epithelial nature of most bladder tumors. Histologically, it is characterized by the proliferation of fibroblastic and myofibroblastic spindle cells accompanied by inflammatory cell infiltration [1]. Although it can occur in almost any anatomical region, it most commonly affects the bladder within the urogenital system and accounts for less than 1% of bladder tumours [2]. The most common presenting symptom of IMT in the bladder is painless macroscopic haematuria; dysuria, urinary frequency, pelvic pain, and obstructive symptoms may also occur. In a systematic review including 182 patients, the mean age was reported as 38.9 ± 16.6 years. Cases have been reported in patients aged three to 89 years [3]. Because IMT has no specific radiological or cystoscopic appearance, it is difficult to distinguish from other bladder tumors. Therefore, the definitive diagnosis is established by histopathological examination and immunohistochemical evaluation [2].

Although the etiopathogenesis is not fully understood, ALK gene rearrangements support neoplastic development, whereas the presence of ALK-negative cases suggests different molecular and inflammatory mechanisms [1,3]. Reports of IMT developing after previous transurethral procedures are among the observations supporting a possible role for chronic inflammation.

Intravesical Bacillus Calmette-Guérin (BCG) exerts an antitumor effect by stimulating the immune response in the treatment of non-muscle-invasive bladder cancer (NMIBC); however, it may also cause marked inflammatory and granulomatous reactions [4]. In this case report, we present a patient who was histopathologically diagnosed with IMT after intravesical BCG therapy for NMIBC. To the best of our knowledge, a similar case of IMT following intravesical BCG therapy has not previously been reported in the literature. The potential relationship between intravesical BCG therapy and IMT development, as well as the importance of considering IMT in the differential diagnosis, is discussed.

## Case presentation

A 74-year-old male patient presented at our clinic with painless macroscopic hematuria. The pathology of the initial transurethral resection of bladder tumor (TURBT), performed in three years earlier, was reported as low-grade urothelial carcinoma (TaLG). Subsequently, no tumor or malignant pathology was detected during the cystoscopies carried out in the third and 12th months. At month 18, cystoscopy revealed multifocal papillary lesions measuring 4-5 mm, and repeat TURBT was performed. After histopathological examination confirmed TaLG, the patient received intravesical BCG induction therapy consisting of six instillations.

Three weeks after completion of BCG induction therapy, the patient again presented with painless macroscopic hematuria. Computed tomography (CT) showed an intravesical mass lesion measuring approximately 120 × 90 mm that largely filled the bladder lumen (Figure 1). ​​​​​​

**Figure 1:**
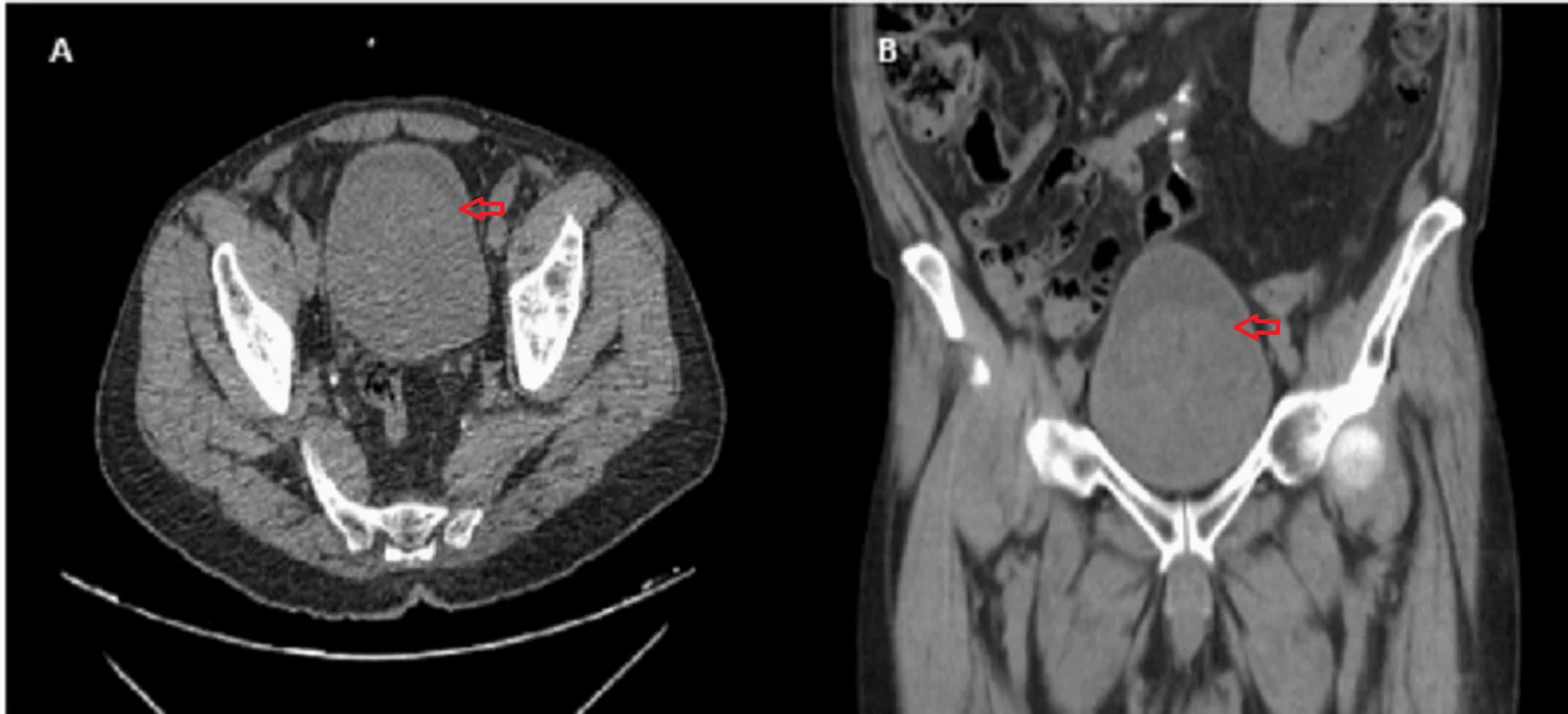
Non-contrast computed tomography images of the bladder lesion. A) Axial non-contrast CT images demonstrating a large intravesical lesion occupying a substantial portion of the bladder lumen. B) Coronal non-contrast CT images demonstrating the same intravesical lesion.

Cystoscopy revealed a solid lesion measuring approximately 120 × 90 mm, filling most of the bladder lumen and differing in morphology from the previous papillary tumors. It originated from the trigone and, apart from a single 10-mm stalk, had no connection to the bladder mucosa; the lesion was resected (Figure 2).

**Figure 2:**
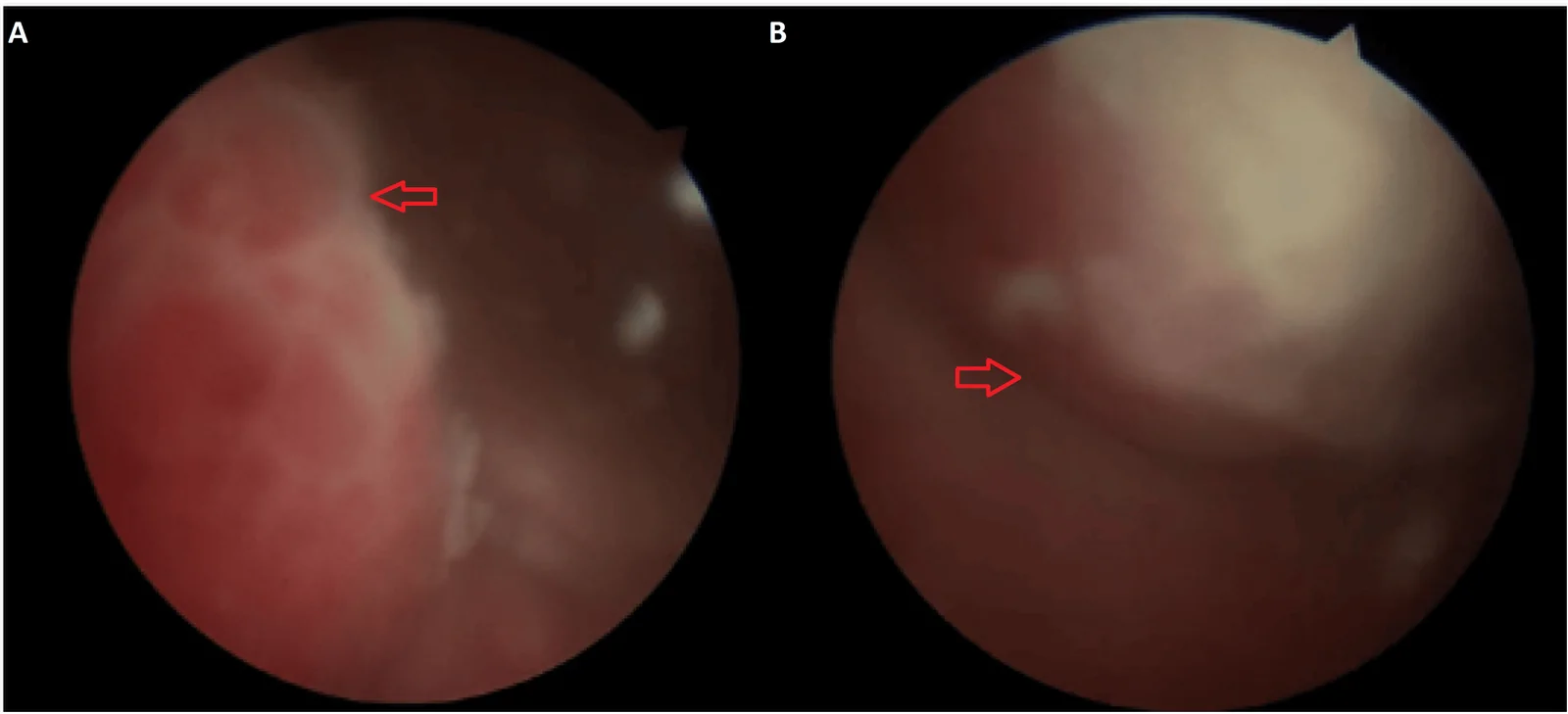
Cystoscopic appearance of the bladder inflammatory myofibroblastic tumor. (A) Cystoscopic view showing a large, smooth-surfaced, non-papillary intravesical lesion. (B) Close-up view demonstrating the pedunculated attachment of the lesion to the bladder wall (arrows).

Based on its endoscopic appearance, the lesion was initially considered to be an organized intravesical hematoma; however, as it did not disintegrate despite irrigation and a mucosal attachment was identified, TURBT was undertaken. Approximately 200 mL of tissue material was obtained during the TURBT. Histopathological examination revealed irregular fascicles of plump spindle cells showing mild cytological atypia and a mitotic activity of 5 per 10 high-power fields, without atypical mitoses or tumor necrosis. Immunohistochemically, the spindle cells were positive for vimentin and focally positive for SMA and MSA, supporting myofibroblastic differentiation, whereas ALK was negative. Negative staining for pancytokeratin, CK7, CK20, p40, p63, GATA3, and EMA argued against sarcomatoid urothelial carcinoma. Desmin, S100, CD34, CD31, CD56, CD99, and β-catenin were also negative, helping to exclude other spindle-cell neoplasms. Considering the morphological and immunohistochemical findings, the lesion was classified as a postoperative spindle-cell nodule within the spectrum of pseudosarcomatous myofibroblastic proliferations and was considered consistent with an ALK-negative IMT (Figure 3).

**Figure 3:**
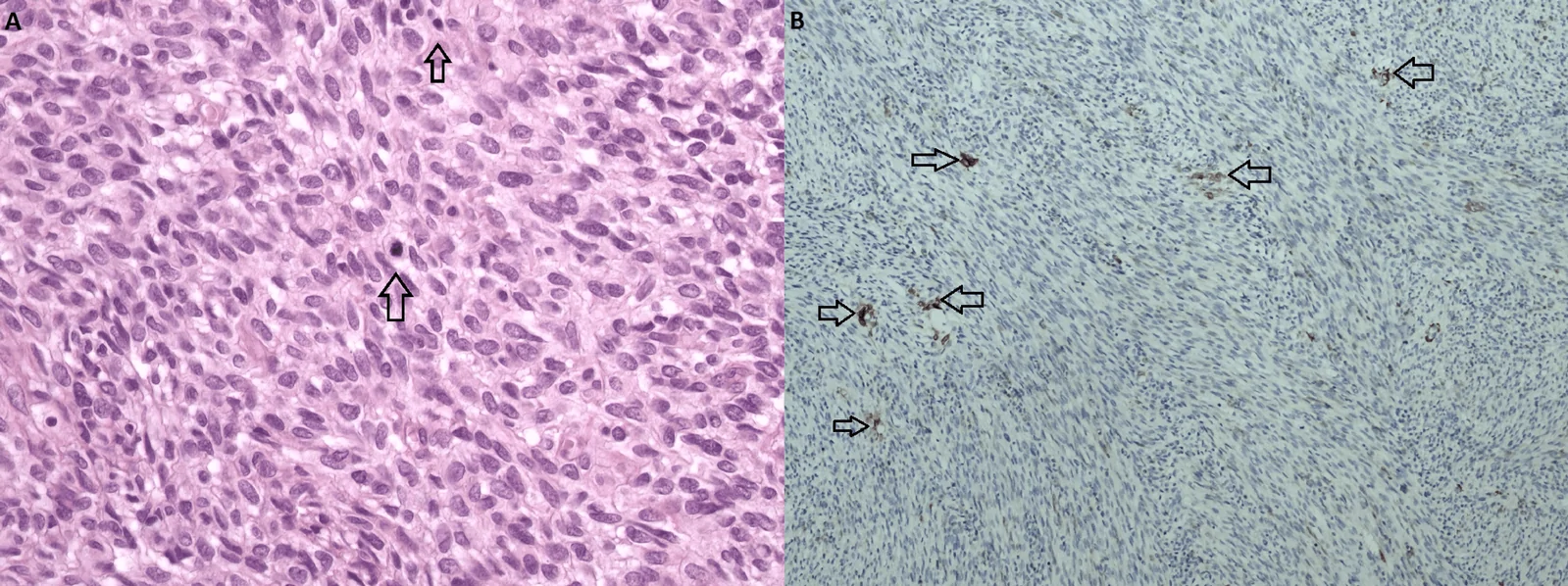
Histopathological findings of inflammatory myofibroblastic tumor. A) Hematoxylin and eosin-stained section showing spindle-cell proliferation with mitotic figures (arrows) (×400). B) Immunohistochemical staining showing focal SMA positivity in spindle cells (arrows) (×100).

The case was reassessed by the multidisciplinary board. Considering the patient's clinical history, endoscopic findings, and pathological diagnosis, the board decided to perform a repeat transurethral resection (re-TURBT). The pathology report of the re-TURBT revealed no residual malignancy. Following this, close cystoscopic surveillance was initiated; the patient underwent cystoscopy at three-month intervals, and no recurrence was detected during the one-year follow-up period.

## Discussion

Although bladder IMT is rare, it is important because it can clinically and endoscopically mimic other bladder tumors. Therefore, the definitive diagnosis is established by histopathological examination and immunohistochemical evaluation [5]. In our case, the fact that the non-papillary lesion measuring 120 × 90 mm, which was detected three weeks after completion of BCG induction and filled a large portion of the bladder lumen, was initially assessed as an organised hematoma illustrates this diagnostic difficulty.

The etiopathogenesis of IMT has not been fully elucidated. Although ALK gene rearrangements support tumor development, ALK negativity does not exclude the diagnosis. Surgical intervention, trauma, and chronic inflammation are also regarded as possible triggers. In the series by Montgomery et al. comprising 46 urinary tract IMTs, a history of recent instrumentation in eight patients supports a possible contribution of tissue injury and the repair process [6]​​​​.

Intravesical BCG induces a strong local immune response in the bladder mucosa, leading to marked inflammation. Histopathologically, it has been demonstrated that chronic inflammation and granulomatous reactions may develop following this treatment [4]. To our knowledge, no previous cases of bladder IMT developing after intravesical BCG therapy have been reported in the literature. The detection of a large mass in the present case just three weeks after the completion of BCG induction therapy suggests a possible temporal association between BCG and IMT. Although causality cannot be established based on a single case, we hypothesize that the intense inflammation and immune response induced by BCG, combined with the mucosal damage from transurethral procedures, may have triggered myofibroblastic proliferation. 

The fundamental approach to bladder IMT is bladder-preserving treatment wherever possible. Systematic reviews have reported that the majority of patients are treated with TURBT or partial cystectomy, while radical cystectomy is performed less frequently [7]. Although recurrence and metastasis have rarely been reported, cystoscopic surveillance is required. In our case, the decision to perform re-TURBT and close cystoscopic follow-up was considered an appropriate bladder-preserving approach. No tumor recurrence was detected during the one-year follow-up. When a non-papillary mass with a morphology different from that of previous tumors is detected following BCG treatment, considering IMT in the differential diagnosis may help to avoid unnecessary radical surgery.

## Conclusions

Bladder IMT is a rare mesenchymal neoplasm that may mimic recurrence of urothelial carcinoma. Although BCG-induced inflammation and immune response may have contributed to the development of IMT in this case, a definitive causal relationship could not be established.

Given the millions of intravesical BCG treatments administered worldwide, IMT may represent an extremely rare adverse event. However, causality cannot be established from a single case. Additional case reports and multicenter studies with long-term follow-up are needed to clarify this possible association.
